# Epidemiology of paracoccidioidomycosis in Venezuela: a retrospective study from 1954 to 2019

**DOI:** 10.1590/0074-02760210203

**Published:** 2021-11-08

**Authors:** Primavera Alvarado, Marcus de Melo Teixeira, Elsy Cavallera, Hugo Costa Paes, Giovanni Guerra, Gerardo Santander, Rommie Merino-Alado

**Affiliations:** 1 Instituto Autónomo de Biomedicina Dr Jacinto Convit, Laboratorio de Micología, Caracas, Miranda, Venezuela; 2 Universidad Central de Venezuela, Facultad de Medicina, Caracas, Miranda, Venezuela; 3 Universidade de Brasília, Faculdade de Medicina, Brasília, DF, Brasil; 4 Universidad Central de Venezuela, Instituto de Geografía Regional, Caracas, Miranda, Venezuela; 5 Universidad Central de Venezuela, Facultad de Odontología, Caracas, Miranda, Venezuela

**Keywords:** paracoccidioidomycosis, epidemiology, diagnosis, Paracoccidioides venezuelensis

## Abstract

**BACKGROUND:**

Paracoccidioidomycosis (PCM) is a systemic mycosis endemic to Latin America. Etiological agents are *Paracoccidioides* species that diverge phylogenetically throughout South America.

**OBJECTIVES:**

This study aimed to document the epidemiology of PCM in Venezuela.

**METHODS:**

We have performed a retrospective cross-sectional descriptive study in 31,081 clinical records of patients from two reference centres during 65 years (1954-2019).

**FINDINGS:**

PCM diagnosis was confirmed in 745 patients. Chronic PCM was the most prevalent form (90.06% cases); 80.67% were male and the most affected age range was 41-60. Farming and construction were the most prevalent occupation and Miranda State had a higher prevalence. Lung and skin were the most affected organs, followed by oral manifestations. Direct examination, culture and serology showed a high sensibility, and no statistical difference was observed among the diagnostic tools. Out of 17 *Paracoccidioides* isolates genotyped from Venezuela, one was typed as *Paracoccidioides americana* and 16 as *Paracoccidioides venezuelensis*.

**MAIN CONCLUSIONS:**

Clinical manifestations observed, information about the epidemiology and molecular profile is essential not only for diagnosis but also for understanding therapeutic responses to mycotic drugs and prognosis. Therefore, it is necessary to sequence all positive isolated strains in order to confirm the dominance of *P. venezuelensis* in Venezuela.

Paracoccidioidomycosis (PCM) is a systemic granulomatous disease restricted to Latin America and mostly reported in humid and tropical areas of Brazil, Colombia, Venezuela, and Argentina.^1^ This mycosis is highly prevalent in rural areas, affecting patients with low socioeconomic status and leading to life-threatening clinical conditions. However, cases have been reported in urban areas linked to constructions, recent urbanisation, and patients with underlying immunosuppression.^2^ ^,^ ^3^ The etiological agents of PCM belong to the genus *Paracoccidioides* in the family *Ajellomycetaceae*, order Onygenales, along with other endemic human pathogens such as *Histoplasma* spp. and *Coccidioides* spp*.* ^4^ *Paracoccidioides* spp*.* are thermally dimorphic fungi that exhibit two morphotypes: a mold occurring at temperatures below 28ºC, composed of thin septate hyphae that might produce conidia (considered as the infectious propagules);^5^ and a yeast form found in cultures or in the host at 37ºC and composed of variably-sised multi-budding oval to round cells.^6^

In recent years, the taxonomy of the genus has changed; before 2006, *Paracoccidioides* only included one species, *Paracoccidioides brasiliensis*.^4^ In 2009, Teixeira et al. reported the phylogenetic distribution of *Paracoccidioides* in a wide range of isolates using multi-locus DNA sequence typing. The authors described a new species named *Paracoccidioides lutzii* that is endemic to Midwestern Brazil, with few cases observed in the Southeast and Ecuador.^7^ ^,^ ^8^ ^,^ ^9^ However, the finding of cryptic species within *P. brasiliensis* drove researchers to explore the evolutionary mechanisms that were responsible for the geographic distribution of four other molecular clades that had been previously characterised in it (S1, PS2, PS3, PS4). Recently, Turissini et al.^10^ performed a study on 65 isolates of *P. brasiliensis* to compare nuclear and mitochondrial DNA polymorphisms and *in vitro* phenotypic variation in isolate morphology. These data led to the establishment of a new classification of the *P. brasiliensis* complex comprised of four new species: *P. brasiliensis sensu stricto* (clades S1a and S1b), *Paracoccidioides americana* (PS2), *Paracoccidioides restrepiensis* (PS3) and *Paracoccidioides venezuelensis* (PS4).^10^ *P. brasiliensis sensu stricto* is widely dispersed in the southern part of South America. *P. americana* (PS2) has been reported in southeast Brazil and rarely in Venezuela, *P. restrepiensis* (PS3) is found predominantly in Colombia and in other Latin American countries and *P. venezuelensis* (PS4) appears to be dominant in Venezuela.^11^

PCM is a pleomorphic disease with diverse clinical manifestations that can lead to misdiagnosis. In 1986, during the *Coloquio Internacional en Paracoccidioidomicosis* (Medellín, Colombia), the disease classification still in use was proposed: acute/subacute, chronic, and residual.^12^ Chronic PCM is the most prevalent clinical form of the disease (up to 74%-96% of the reports), characterised by pulmonary and oral lesions due to lymphohematogenous dissemination of the pathogen. Most patients are male and lesions are usually deep and painful granulomatous-like lesions; pulmonary insufficiency can be reported in these cases. Lesions can be found in the mouth, intestines, adrenal glands, central nervous system, lymph nodes and bones. In contrast, the acute/sub-acute form is frequently observed in infants and adolescents, representing 5% to 25% of reported cases and featuring hepatosplenomegaly and severe lymphadenopathy.^13^ ^,^ ^14^

In Venezuela, the first cases of PCM were reported by O’Daly (1937)^15^ and Guerra (1948), who addressed the importance of studying the lung manifestations of the illness.^16^ In 1971, Albornoz performed the first isolation of the fungus from soil samples collected at the Miranda State^17^ and coupled with some evidence of high positivity of intradermic surveys in the area. In the past 30 years, Venezuela has gained prominence in the PCM epidemiological roadmap due to the high incidence in the North part of the country as well as in the Bolivar State, which is characterised by tropical rainforest.^18^ This places Venezuela third in the numbers of reported cases of this deep mycosis, in association with a unique *Paracoccidioides* species. Therefore, this study aimed to characterise the clinical and epidemiological aspects of the PCM caused by *Paracoccidioides* species in Venezuela.

## MATERIALS AND METHODS

*Study design* - We conducted a retrospective, cross-sectional and descriptive study by evaluating patients’ medical records referred to the Dermatology Department of the Hospital Vargas, Instituto Autónomo de Biomedicina “Dr. Jacinto Convit”, Laboratorio de Micología. Caracas, Venezuela and Microbiology Department of the Dentistry School, Universidad Central de Venezuela (UCV). We reviewed 31,081 clinical records of patients in a 65-year period (from 1954 to 2019). Data from Dentistry School were available from 2016-2019. All centres received patients and samples from the Metropolitan area and from outside the capital region.

*Data collection* - Data collected included demographic, epidemiological, clinical and radiographic findings of the PCM cases observed in patients who attend the medical centers as mentioned above.

*Ethical statements* - Patients’ data were included after ethical consent using an anonymous instrument previously approved by the Ethics Committee Board of the Instituto Autónomo de Biomedicina “Dr. Jacinto Convit” (number 08/08/2016).

*Laboratory diagnosis* - PCM diagnoses were confirmed by either direct examination, microbiological isolation or serological tests. Direct examinations were performed from biopsies and stained with 40% KOH and Giemsa stain. For the microbial isolation of *Paracoccidioides* sp., the affected tissues were cultured in Sabouraud Dextrose Agar (SDA), with chloramphenicol, and incubated at 25ºC and 37ºC for 60 days. Macro - and microscopic characteristics compatible with *Paracoccidioides* sp. were searched. Later, all positive cultures were stored in distilled water according to the Castellani method and recovered for additional analysis after new subcultures on SDA with chloramphenicol.^19^ After 1975, a double immunodiffusion assay (serology) was implemented as part of the diagnosis algorithm in our centre. The Double immunodiffusion assay (DID) was performed for specific antibody detection, using *P. brasiliensis* antigen obtained from the Pb339 strain.^20^ All patients’ sera were collected before PCM treatment and then in repeated drawings for the disease monitoring during a 6-month period. In addition, serum titration was performed in all samples through serial dilutions in the course of diagnosis and treatment.

*Molecular epidemiology* - We evaluated 17 Venezuelan *Paracoccidioides* sp. strains genotyped so far using different research approaches by either restriction fragment length polymorphism (RFLP), rapid amplified DNA polymorphisms (RAPD), multi locus sequencing typing (MLST), or by whole genome typing (WST). In addition, the genotype and approximate location (to state level) were retrieved from previous studies.^4^ ^,^ ^11^ ^,^ ^21^

*Statistical analysis* - The patients’ epidemiological and clinical data were represented as frequencies and their respective 95% confidence intervals were calculated. The Fisher exact test was also performed to analyse contingency tables by comparing clinical forms and affected organs.

The diagnostic tests were compared using *N-1* Pearson’s Chi-Square test with 95% confidence intervals (95% CI). In addition, squared chi person *N-1* was applied to determine statistical differences between groups. A *p-value* below 0.05 was considered statistically significant.^22^ The classical quantitative variables, sensitivity and specificity, were determined with indicators oscillating between 0 and 100%. The Pearson chi-squared test was employed to establish whether statistically significant differences existed between these variables.^23^

## RESULTS

**Fig. 1:**
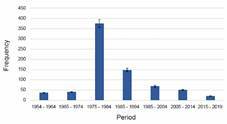
temporal distribution of paracoccidioidomycosis (PCM) cases in Venezuela from 1954 to 2019.

We retrospectively reviewed 31,081 medical records from 1954 to 2019; 745 (2.39%) patients had PCM diagnosis confirmed by either direct examination, microbiological isolation, or serology. 601/745 (80.67%) were males and 144/745 were females (19.32%), a 4:1 male-to-female proportion. The age range was from 7 to 88 years and the mean age of affected individuals was 49.5 years (Table I). Regarding occupation, 242 (32.48%) were rural workers, followed by other professions such as construction workers, general maintenance workers (32.34%), builders (12.34%), domestic service workers (9.12%), among others (Table II). The distribution of diagnosed cases showed that most were in the 1975-1984 period, representing 52.01% of all diagnoses (Fig. 1).

**TABLE I:** Age range by gender of patients with paracoccidioidomycosis (PCM) in Venezuela from 1954 to 2019

Age range (years)	Female	Male	Total	Ratio
< 10	-	11	11	-
11 - 20	-	17	17	-
21 - 30	2	37	39	18.5
31 - 40	7	69	76	9.9
41 - 50	25	221	246	8.8
51 - 60	77	152	229	2
61 - 70	24	64	88	2.6
> 70	9	30	39	3.3
Total	144	601	745	4.17

**TABLE II:** Demography and epidemiological of patients with paracoccidioidomycosis (PCM) in Venezuela from 1954 to 2019

Variables	N	Percentage (%)
Gender		
Male	601	80.67
Female	144	19.32
Total	745	100
Occupation		
Farmer	242	32.48
General service worker	241	32.34
Builder	92	12.34
Housekeeping	68	9.12
Merchant	47	6.30
Student	30	4.02
Ground transportation	12	1.61
Electric technicians	9	1.20
Engineers	2	0.26
Teacher	2	0.26
Total	745	100

**Fig. 2:**
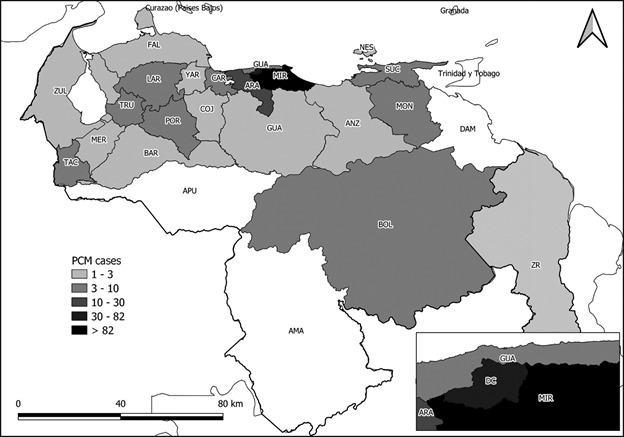
geographic distribution of paracoccidioidomycosis (PCM) in Venezuela from 1954 to 2019. The states are abbreviated as follows: Amazonas (AMA), Anzoátegui (ANZ), Apure (APU), Aragua (ARA), Barinas (BAR), Bolívar (BOL), Carabobo (CAR), Cojedes (COJ), Delta Amacuro (DAM), Distrito Capital (DC), Falcón (FAL), Guárico (GUA), Lara (LAR), Mérida (MER), Miranda (MIR), Monagas (MON), Nueva Esparta (NES), Portuguesa (POR), Sucre (SUC), Táchira (TAC), Trujillo (TRU), La Guaira (GUA), Yaracuy (YAR), Zulia (ZUL). Base map obtained from the Simón Bolívar Geographical Institute of Venezuela (IGVSB). Data obtained from the Mycology Laboratory of the Autonomous Service Institute of Biomedicine “Dr Jacinto Convit”.

We observed patients with PCM from all over the country, but most came from Miranda (75.03%), Distrito Capital (12.61%) and Aragua (3.08%), located in the northeastern portion of the country (Fig. 2). Miranda State cases were overrepresented in a 10:3 ratio to all the other states, which is far more than its historical proportion of the population; Miranda State is located in the northern portion of the country and most of the cases were reported in rural areas compared to urban or outermost regions.

Regarding the clinical manifestations of PCM, the chronic form was the most prevalent and observed in 90.06% of the patients, a proportion that was not skewed by gender. On the other hand, the acute form only represented 9.93% of the reported cases (Table III). The most affected organs associated with chronic forms were lung (100%), skin (43.22%), followed by oral mucosa (28.32%) and nose (10.60%); other organs were affected in 8.32% cases (Table IV).

**TABLE III:** Clinical forms of paracoccidioidomycosis (PCM) in Venezuela and its correlation with comorbidities and patient gender from 1954 to 2019

	Female		Male		Total		
	N = 144	%^*a*^	N = 601	%^*a*^	N = 745	%^*a*^	p-value^*b*^
Clinical forms							
Acute/sub-acute	7	4.86	67	11.14	74	9.93	0.008
Chronic	137	95.13	534	88.85	671	90.06	0.008
Comorbidity							
Carcinomas	0	0	4	0.66	4	0.53	0.478
HIV/AIDS	3	2.08	16	2.66	19	2.55	0.913
Histoplasmosis	0	0	4	0.66	4	0.53	0.478
Tuberculosis	0	0	1	0.16	1	0.13	1.000

*a*: proportion value based on calculations through contingency tables; *b*: p-value in a two-sample z test for the difference of proportions.

**TABLE IV:** Clinical forms of paracoccidioidomycosis (PCM) in Venezuela and affected organs

	Acute/sub-acute		Chronic		Total		
Affected organ	N = 74	%^*a*^	N = 671	%^*a*^	N = 745	%^*a*^	p-value^*b*^
Oral mucosa	0	0	211	31.44	211	28.32	< 0,0001
Nose	0	0	79	11.77	79	10.60	< 0,0001
Lymph nodes	61	82.43	2	0.29	63	8.45	< 0,0001
Eyes	1	1.35	7	1.04	8	1.07	1,000
Skin	0	0	322	43.22	322	43.22	< 0,0001
Lung	74	0	671	100	745	100	< 0,0001
Other organs^*^	12	16.22	50	7.45	62	8.32	0,069

*a*: proportion value based on calculations through contingency tables; *b*: p-value in a two-sample z test for the difference of proportions; *: skin, larynx, and central nervous system.

**TABLE V:** Laboratory diagnosis of paracoccidioidomycosis (PCM) from 1954 to 2019 in Venezuela

Test	Negative	%	Positive	%	Total
Direct examination	196	26.30	549	73.69	745
Conventional culture	102	13.69	643	86.30	745
Serology (IDD)^*^	67	11.07	502	88.22	569

*: IDD was available from 1975.

The main manifestations associated with the clinical forms of PCM were fever, observed in 72.7% of acute/subacute cases and 27.3% of chronic cases; weight loss was also observed in 70% of all studied cases. Pulmonary infiltrates were observed in all patients with the chronic form. In addition, patients diagnosed with acute/subacute PCM had generalised lymphadenopathy (95.6%), splenomegaly (44.2%) and hepatomegaly (35.6%). In PCM patients with the chronic form, the lesions were primarily located in the skin in 47.98 % of the patients and oral mucosa involvement was observed in 31.44% of cases (Table IV).

The most frequent comorbidity was HIV/AIDS, found in 19 of the 745 patients (2.55%). We observed four patients co-infected with histoplasmosis and four reported carcinomas; one patient had tuberculosis combined with a chronic multifocal form of PCM (Table III). The diagnosis was conﬁrmed using three techniques: direct microscopic examination of various patient samples (obtained by biopsy, sputum and exudate aspiration), culture and double immunodiffusion (Table V). Conventional culture had 86.30% sensitivity (95% CI = 84% - 88%) and colonies were obtained in 14 to 60 days. DID had 88.22% (95% CI= 86% - 91%) and the serum titration average of our cohort was between 1:16 and 1:64. Sensitivity of direct examination was 73.69% (95% CI = 71% - 77%). Pearson’s Chi-Square statistical analysis of diagnosis tools sensitivity did not show a significant difference among the three techniques.

Molecular epidemiology of PCM in Venezuela

In our study, we identified 17 *Paracoccidioides* clinical isolated strains from Venezuela. All genotyping methods are congruent and suggest strong geographical isolation of a particular species of *Paracoccidioides* in Venezuela. Sixteen out of seventeen strains of *Paracoccidioide*s were identified as *P. venezuelensis*; a single strain was typed as *P. americana* (PS2).

## DISCUSSION

PCM is a systemic mycosis restricted to Latin America with a wide range of clinical manifestations that can affect any susceptible host and is considered endemic in Brazil, Venezuela, Colombia, Ecuador and Argentina. For example, Chile has no reports of autochthonous cases and this can be associated with a specific niche for fungal development.^24^ In this sense, the climate characteristic of Venezuela creates appropriate conditions for not only *Paracoccidioides* spp., but other dimorphic fungi that cause systemic mycoses such as *Histoplasma* spp*.* and *Coccidioides posadasii*. Thus, it is crucial to consider these agents as a differential diagnosis in granulomatous-like diseases.^25^ The *reservarea* suitable for the development of the *Paracoccidioides* spp. saprophytic phase in Venezuela, defined by Borelli, is characterised by tropical and subtropical areas surrounded by the Andes, San Luis mountains and the Macizo Guayanes, with a temperature range of 10ºC to 28ºC, 500 to 2500 mm/per year pluviosity, an altitude ranging from 47 to 1,300 meters, acidic soil pH and abundant waterways.^26^ ^,^ ^27^ ^,^ ^28^

Our study depicted the epidemiology of PCM in Venezuela with 745 cases out of 31,081 medical charges in 65 years, all of them diagnosed in the Distrito Capital area. The clinical cases of PCM herein described and the recent reports of this disease allow us to confirm that Venezuela is the third most affected country with PCM in Latin America. However, it is estimated a significant underreporting throughout Latin America, and the number of PCM cases described in Colombia and Argentina, countries of high PCM incidence, could be also underestimated.^29^

Time-lapse distribution showed a higher prevalence between 1975 and 1984 with 52.01% of PCM diagnosed cases. After 1984 the patients’ flow to the capital region decreased, which can be attributed to the deployment of well-trained personnel and the establishment of PCM workgroups around the country to achieve diagnosis outside the metropolitan area. In more recent times, roughly from 2005 onwards, the emigration of skilled professionals has meant that the scarce personnel left is mainly located in the Distrito Capital. The remaining working groups are liaising to improve the diagnosis and collect accurate data of PCM and other mycoses in Venezuela.

Regarding the geographic distribution, 75.03% of our cases were from Miranda State, a highly endemic region for PCM, followed by Distrito Capital with 12.61% and Aragua with 3.08%. It is well-known that the north portion of the country has accounted for the highest number of cases; however, the epidemiological trends are evolving so that the entire country can be considered endemic to PCM. It has been reported that PCM is endemic in Carabobo, Lara, Monagas and Aragua states; throughout the years, our workgroup in Distrito Capital has received patients from all over the country. Those observations allow us to hypothesise a high rate of miss-notification and sub-diagnosis across Venezuela, such as in the Delta Amacuro, Apure and Amazonas states.^30^

About demographic features, 80.67% of patients were males in a 4:1 ratio in comparison with female affection, as previously reported in other endemic countries such as Brazil (93.4%), Colombia (80%) and Argentina (70%); in Brazil, there is a wide range of male-female ratio reported from 3:1 to 10:1 according to the population studied.^31^ ^,^ ^32^ ^,^ ^33^ ^,^ ^34^ In Venezuela, highly endemic areas such as Miranda can have a higher male-female proportion. Another study cohort with Venezuelan patients reports a similar male-female ratio 6,5:1 in the studied population.^28^ Although PCM is more prevalent in males, females are susceptible to acquiring or developing the infection previously to menarche or post-menopause due to the interaction between estrogens and *Paracoccidioides* spp.^35^

This mycosis has been associated with farming, a traditionally male activity; nevertheless, data of PCM cases in women is increasing. In a recent 2020 study, a group of Brazilian women between 40 and 50 years was affected and the social history of patients revealed to be housewives and rural workers with systemic changes at the time of PCM diagnosis, namely: pregnancy, HIV infection and/or depression. All these factors are related to hormonal impairment, contact with a massive inoculum size as observed in traditionally male activities such as farming, or immunological alterations that affect cellular response in the female hosts.^36^ Of note, our series showed 21 cases of chronic form in adult women younger than 48 and one more case which had HIV coinfection; this indicates that the distribution of PCM clinical cases is in fact much more skewed than evident by a plain male: female ratio, as age stratified ratios on Table IV highlight. We suggest that female cases of PCM in reproductive age should be treated with special care, as it cannot be excluded that factors related to the fungus itself may influence the occurrence of non-climacteric, adult female cases.

In our study, 33.02% of the patients diagnosed were in the 41-51 age range; there is copious literature that reports PCM infection in patients from 2 to 102 years old, with most cases being reported in patients over 30.^14^ ^,^ ^37^ Immunosenescence plays a role in the development of PCM in elderly patients, along with the interaction of hormone receptors previously discussed.^35^ Our data showed that the more susceptible occupations to acquire and develop PCM were farmers (32.48%) and others with substantial exposure risk (manual workers, builders; 32.34%), which might be related to a high inoculum of conidia. In both cases, conidia are inhaled by the direct contact with soil and dust originated from their activities.^38^ ^,^ ^39^ In the last decade, a substantial fraction of the low-income Venezuelan population has turned to agriculture as a response to a profound economic shortage: in search of a profitable side job, people go into farming of sugarcane, coffee, rice, cotton, corn and other crops. Additionally, migration of rural population into urban areas, job shifting and the fact that this mycosis is diagnosed many years after primo-infection have generated a scenario where the disease does not correlate strongly with the patients’ occupation and the location of cases in medical registers of the evaluated patients.^28^

Regarding clinical forms of PCM, our study showed that patients between 30 to 60 years old developed chronic disseminated PCM with 90.06% of the clinical manifestation. Only 9.93% of cases were classified as acute/juvenile forms. Chronic PCM presentation is the most prevalent clinical form of the disease and is widely reported in adult and elderly patients in other countries in South America;^14^ it can be unifocal or multifocal according to the number of organs affected in hosts. Chronic PCM can lead to a wide range of non-specific systemic symptoms and mimic other granulomatous diseases, and diagnosis is challenging.^14^ ^,^ ^40^ Regarding the organs affected in the patient metadata, the skin was the most reported (43.22%), followed by oral mucosa (28.32%); 8.32% of patients showed disease in other sites, including nose, larynx, and central nervous system. It is well documented that the chronic form is the most prevalent among male patients and usually affects oral mucosa, upper and lower respiratory tracts. Therefore, skin and oral manifestations, along with high suspicion due to epidemiological information, must be a clue for physicians to suspect PCM infections.^14^ ^,^ ^31^ ^,^ ^41^

As for comorbidities, 19 (2.55%) of our patients had HIV/AIDS with PCM diagnosed after 1989, which concurs with the emergence of the AIDS pandemic; literature refers that 5% of the patients with HIV/AIDS can develop PCM. However, the correlation between HIV/AIDS and PCM is slowly decreasing due to the early initiation of antiretroviral treatment in these patients.^42^ In addition, four patients had concurrent neoplasms, four had histoplasmosis, and only one had TB coinfection. Previously, authors have established the association between TB and PCM, ranging from 15% to 20% of incidence.^43^ Other reports from Venezuela had shown a higher concurrence of PCM and histoplasmosis, representing over 28% of the studied cases.^44^ Authors such as Shikanai-Yasuda previously report carcinoma concurrence in 0.16% to 14.1% of patients during PCM infection or after therapeutic management of PCM. In any case, a reliable and interdisciplinary study of clinical manifestations is mandatory to rapidly discard any malignancy associated with PCM lesions.^45^

About mycological diagnosis, three techniques were assessed to accomplish the diagnosis: DID with 88.22%, followed by conventional culture with 86.30% and direct examination with 73.69% of sensitivity, respectively. Pearson’s Chi-Square Statistical analysis of diagnosis tools sensitivity did not show significant difference among the three techniques, which leads us to state that any of the tools can satisfactorily accomplish the diagnosis by well-trained personnel and that having more than one test available can improve the quality and increase the speed of diagnosis. It must be noted, though, that authors such as Sylvestre et al. in 2014 quoted a high sensitivity of serology up to 80% after antifungal treatment^20^ so past disease may be a confounding factor when trying to find the cause of a mucocutaneous lesion and PCM is suspected.

Previous studies have reported that culture in SDA is a gold standard in the diagnosis algorithm of PCM; however, *Paracoccidioides* spp. is a slow-growing fungus, which affects the sensitivity of conventional culture methods.^46^ In contrast, other authors such as Hahn et al.^47^ report a high sensitivity in mycological culture (97%) and direct examination (88.2%) in *P. lutzii*. It is worth pointing out that in our case series, from 1954 through 1974, direct examination and mycological culture were the only available methods to PCM diagnosis, and DID was included as a complementary diagnostic tool after 1975.

In our study, we included 745 isolated strains from clinical samples in a 65-years period; most of the previously collected strains were stored according to Castellani’s method. After a variable period (1 to 65 years), only 56 (8.7%) strains were successfully recovered, and 17/56 were qualified as appropriate for the molecular study.^11^ *Paracoccidioides* spp. culture and storage are still a challenge; conventional storage methods are Castellani’s technique or mineral oil conservation. Previous authors report a 26% recovery rate of the collected strains onto fresh culture media, with a limit of ten years of storage.^48^

Our study documents the epidemiological history of PCM in Venezuela and is the third-largest case series after studies from Colombia and Brazil.^14^ ^,^ ^29^ ^,^ ^38^ Molecular epidemiological studies carried out so far in Venezuela suggest that *P. venezuelensis* (or PS4) is the main etiological agent of PCM, which our study seems to confirm. Genomic analysis indicates that *P. venezuelensis* represents a unique genotype in the *Paracoccidioides* genus and shares a common ancestor with *P. restrepiensis*, which is endemic to Colombia.^11^

This retrospective study allowed us to establish an epidemiological footprint of PCM in Venezuela during a 65-year period, confirming the significant endemicity of this mycosis in our country. Comprehension and knowledge of clinical, epidemiological and genomic data can help us implement an adequate disease follow-up. It is important to highlight that this mycosis is not considered a mandatory notification disease despite being linked to a significant rate of morbimortality in underdeveloped countries such as Venezuela.
